# Effects of apparent temperature on cardiovascular disease admissions in rural areas of Linxia Hui Autonomous Prefecture

**DOI:** 10.1038/s41598-023-42232-9

**Published:** 2023-09-11

**Authors:** Guangyu Zhai, Ziyao Gao, Wenjuan Zhou

**Affiliations:** 1 School of Economics and Management, Lanzhou University of Technology, Lanzhou, 730050 People’s Republic of China; 2 Gansu Provincial Hospital, Lanzhou, 730000 Gansu People’s Republic of China

**Keywords:** Cardiovascular diseases, Climate-change impacts, Risk factors

## Abstract

Cardiovascular disease (CVD) is a major threat to public health worldwide. The relationship between CVD and temperature has been widely reported in developed countries and regions. However, there are few studies of severe CVD in poor rural areas of developing countries. Therefore, this study aimed to explore the relationship between CVD and apparent temperature (AT) in a rural area of Linxia Hui Autonomous Prefecture, China. Daily CVD admission data and meteorological data were collected from Linxia between 2014 and 2015. The media of AT was used as the reference temperature to estimate the cumulative relative risk (RR) of CVD admission. The distributed lag non-linear models were used to examine the association between AT and cumulative RR of CVD admission at lag 0–21 days. In Linxia, high AT (20 °C) had a persistent adverse effect on cumulative RR of CVD admissions, and the RR increased with increasing lag days. Cold (− 10 °C) had a protective effect on the first and later lag days (lag 0–14 and lag 0–21). Adults (aged < 65 years) and females were more susceptible to the effects of heat than males and the elderly (aged ≥ 65 years). In Linxia, China, extremely high AT is an important risk factor for CVD hospitalizations in suburban and rural populations.

## Introduction

Cardiovascular disease (CVD) is the most important threat to human health worldwide. Since 2006, there have been approximately 290 million CVD patients in China, and 40% of the deaths are due to CVD, which is higher than the mortality caused by cancer and other diseases. Moreover, since 2009, the mortality rate of CVD in rural areas has exceeded that in urban areas^1^. CVD is also the leading cause of death worldwide, with an estimated 17.7 million deaths in 2015. The economic burden caused by CVD is also huge, and CVD is known as the most expensive disease^2^. The burden of CVD is heaviest in middle-income European Society of Cardiology member countries, where the estimated incidence is 30% higher than that in high-income countries^3^. Thus, the effect of CVD is more severe in low- and middle-income countries. However, as a developing country, China has a significant burden of CVD on public health, especially in rural areas.

Over the years, many studies have found that climate change is an important cause of CVD morbidity and mortality^4–6^. Climate change, especially heat exposure, has various negative effects on cardiac health^5^. Most of these studies have been conducted in developed countries or cities, but few have been conducted in low-and middle-income countries and poor areas, where the CVD burden is more severe. Many studies have used environmental temperature as an indicator to explore the effect of climate on CVD admissions^7–9^. For example, Guo et al.^10^ found that low temperatures had a strong effect on stroke incidence in Guangzhou. Some studies have also investigated the association between weather and CVD admissions using the diurnal temperature range as an indicator^11,12^. However, both temperature and diurnal temperature range are only single temperature indicators, which are inappropriate for the true relationship. Therefore, in this study, we used the composite index of temperature and other meteorological factors to explore the effect of climate on CVD; the composite index of temperature can more objectively reflect the body’s cold and heat perception^13^.

In China, most studies on the relationship between temperature and CVD have been conducted in developed cities, while few studies have been conducted in Northwest rural areas, which are poor and inhabited by ethnic minorities^10,14,15^. Therefore, it is necessary to study the relationship between the cumulative relative risk (RR) of CVD admission and apparent temperature (AT) in rural areas. To this end, we conducted a study to analyze the relationship between extreme AT and cumulative RR of CVD admission in Linxia Hui Autonomous Prefecture between January 2014 and December 2015. These findings can be used to assess the effect of climate change on the health of specific populations, rationally allocate health resources, and prepare for the effect of extreme climate on the population in advance.

## Materials and methods

### Study area

**Figure 1:**
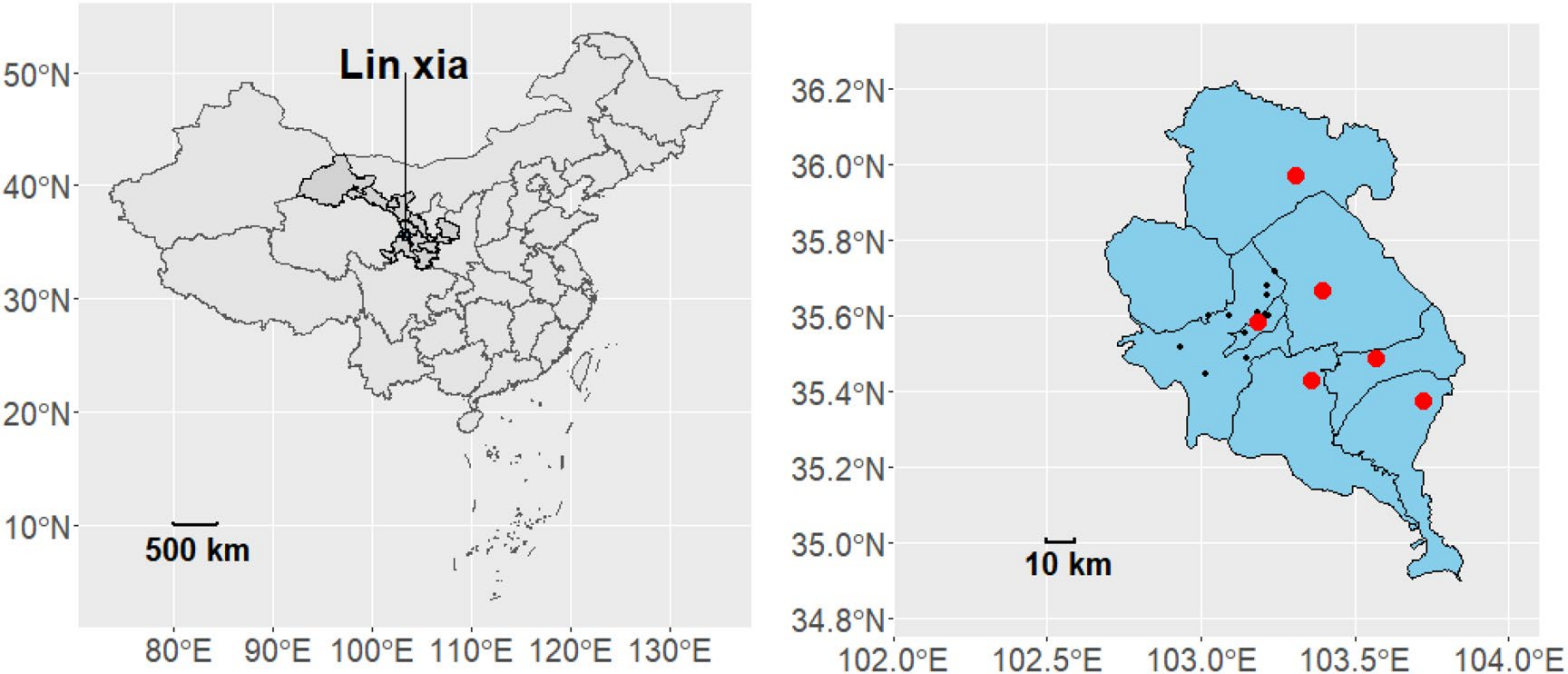
Geographical location of Linxia, Gansu Province, China. In the left map, the shaded area in dark gray represents Gansu Province, while the blue area represents Linxia Prefecture. In the right map, the blue area represents Linxia Prefecture, the red dots indicate meteorological observation stations, and the black dots represent healthcare facilities.

Linxia Autonomous Prefecture (Fig. 1) is located in the Southwest of Central Gansu Province, Northwest of China, at latitude 34° 57′–36° 12′ N and longitude 102° 41′–103° 40′ E. It is located in the transition zone from Qinghai-Tibet Plateau to the Loess Plateau. There are many valleys in the prefecture, and a small number of plains are distributed in Linxia City and Hezheng County. The terrain gradually increases from Northeast to Southwest, showing that the Southwest is high and the Northeast is low, with a tilted basin state. The average altitude is 2000 m, the highest is 4485 m, and the lowest is 1556 m. Linxia Prefecture mainly has temperate semi-arid climate, which can be divided into three climate zones, specifically manifested as high cold and wet in the Southwest mountainous area, dry in the northeast, and mild in the river valley^16^. Therefore, the residents of rural areas experience greater temperature changes, which lead to a higher risk of CVD. In addition, because of the poor economic situation of Linxia, alleviating the poverty of its residents is a difficult task.

### Data collection

We collected CVD hospitalization and meteorological data. The data on CVD hospital admission between January 1, 2014, and December 19, 2015, were collected from the New Rural Cooperative Medical System of Gansu Province, which is a government agency responsible for data collection in Gansu Province. The Gansu Province New Rural Cooperative Medical System was initiated in 2003. By 2007, it had been implemented in 38 counties, covering a rural population of 2,017,7000 people, with a participation rate as high as 88.43%. The implementation of the new rural cooperative medical system in Gansu has greatly helped and improved the healthcare situation for farmers in impoverished areas, effectively curbing the phenomenon of poverty caused by illness in rural areas^17^. The data included age, hospital, sex, residential address, disease, and diagnostic ICD codes.

The meteorological data (2014–2015) were obtained from the Gansu Meteorological Bureau, including wind speed, temperature, local pressure, relative humidity, rainfall, and sunshine duration. The following formulas^13^ were used for the calculation of AT:

$$AT=Ta+0.33\times e-0.70\times WS-4.00$$

$$e=\frac{Rh}{100}\times 6.105\times exp\left(17.27\times \frac{Ta}{237.7+Ta}\right)$$

In the formulas, Ta denotes ambient temperature (°C), e denotes water vapor pressure, WS denotes wind speed (m/s), and Rh is relative humidity (%).

### Statistical analysis

Previous studies have identified a non-linear relationship between AT and CVD and a lagged effect of AT on CVD^18^. Therefore, we used a distributed lag non-linear model (DLNM), a modeling framework that can simultaneously represent non-linear exposure–response dependencies and delayed effects^13,19^. In this model, potential confounders (temperature, relative humidity, sunshine duration, and day of week) were controlled. The following formula was used for the model:

$$\mathit{Log}\left[E\left({Y}_{t}\right)\right]=\alpha +\beta \left({AT}_{t,l}\right)+ns\left(Time,7\right)+ns\left({Sun}_{t},3\right)+ns\left(Rh,3\right)+DOW+Holiday$$

where $E\left({Y}_{t}\right)$ is the daily number of hospitalizations for CVD on day t; t is the day of observation (t = 1, 2, 3 …21); α is the intercept; β is the vector of the coefficients for ${AT}_{t,l}$; ${AT}_{t,l}$ is the cross-basis matrix of DLNM, with the cross-basis function including the exposure–response and lag-response functions; l is the lag days; and $ns$ is the natural cubic spline function to control potential confounders. $Time$ is the long-term tendency with 7 degrees of freedom (df)^20^; ${Sun}_{t}$ is the sunshine duration on day t with 3 df^21^; $Rh$ is the relative humidity with 3 df^21,22^; and $DOW$ and $Holiday$ were dummy variables, which were used for controlling the effects of the day of week and holiday. Previous studies have mentioned the impact of pollutants on cardiovascular diseases^21^. However, in remote rural areas, air pollution is not significant^22^. We conducted a sensitivity analysis on air pollution and no differences were observed (see Fig S1). Therefore, to prevent overfitting, we did not include pollutants in our model. In the cross-basis functions, the natural cubic spline functions was used to define the exposure–response and -lag dimensions. We set spline knots at equally spaced values within the AT ranges for exposure–response dimension and three equally spaced knots along its logarithmic scale for lagged dimension.

Akaike’s information criterion was used to select the fitting df for AT and lag^23,24^. We initially selected 6 degrees of freedom for exposure–response dimension and 5 degrees of freedom for lag-response dimension (see Fig S2). However, the results indicated overfitting of the model. To address this issue, we explored different combinations of degrees of freedom and found that using 4 degrees of freedom for the exposure response dimension and 3 degrees of freedom for the lag response dimension resulted in a better fit of the mode^25^.

The extremely high and low AT were set to 20 °C (95th percentile) and − 10 °C (5th percentile) in the AT distribution. We also performed subgroup analyses for age (< 65 or ≥ 65 years) and gender (male or female), as well as evaluated the effects of different lag days and high and low AT in each subgroup. The median AT (7.47 °C) was used as the reference value to calculate the RR with a 95% confidence interval (CI) and *p* value of 0.05 ^10,26^.

The sensitivity analyses were performed to test the robustness of the model; we changed the df of time from 4 to 12, relative humidity from 2 to 4, and sunshine duration from 2 to 4. We also changed the maximum lag days from 14 to 28. In addition, all data analyses were performed using R software version 4.2.1.

### Ethics approval and consent to participate

All experimental protocols in the study were approved by the ethics committee of Lanzhou University of Technology, all methods were carried out in accordance with relevant guidelines and regulations.

### Consent to methods

I confirm that all methods and procedures were executed in strict accordance with the relevant guidelines and regulations.

### Consent to participate

Informed consent was obtained from all individual participants included in the study.

## Results

There were 15,642 CVD patients between January 2014 and December 2015, comprising 6654 males (42.5%) and 8988 females (57.5%); 8390 old individuals (53.6%) and 7252 adults (46.4%). Table 1 shows the CVD hospitalizations and weather data in Linxia Autonomous Prefecture. The mean daily total hospital admissions were 21.9. The number of CVD cases was higher in females than in males and in the older population than in adults. The mean AT was 6.14 °C, with a range of − 13.82 °C to 24.66 °C, while the mean temperature was 8.49 °C, with a range of − 9.5 °C to 24 °C.

**Table 1:** Summary statistics for daily weather variables and cardiovascular disease (CVD) cases in Linxia Autonomous Prefecture, China, 2014–2015.

	Mean	SD	0%	25%	50%	75%	100%
V1	21.91	10.18	4	16	20.5	27	166
V1 male	9.32	4.87	1	6	9	12	74
V1 female	12.59	6.41	1	8	12	16	92
V1 adult (< 65)	10.16	5.55	1	6	10	13	86
V1 elderly (≥ 65)	11.75	5.67	2	8	11	15	80
Tt (°C)	8.49	8.66	− 9.5	0.9	10.05	16.08	24
AT (°C)	6.14	10.02	− 13.82	− 2.9	7.47	15.06	24.66
Rh (%)	61.97	15.6	20	51	64	73	96
P	807.97	4.1	794	805.2	807.9	810.5	820.4
Rainfall	1.18	3.59	0	0	0	0.3	37.2
Wind speed	1.41	0.5	0.4	1.1	1.3	1.6	3.8
Sunshine	6.3	3.93	0	3.03	7.2	9.4	13.2

V1: total hospital admission; V1male: number of male hospitalizations; V1female: number of female hospitalizations; V1adult: number of adult hospitalizations, V1elderly: number of elderly hospitalizations; Tt: temperature; AT: apparent temperature; Rh: relative humidity; P: atmospheric pressure.

The cumulative RR of CVD hospital admission for the total group at extremely low and high AT during the lag days is shown in Table 2. At extremely low AT (− 10 °C), the RR of hospital admission increased at lag 0 and lag 0–3 and then decreased significantly at lag 0–7, lag 0–14, and lag 0–21, compared with the reference value (the median of AT, i.e., 7.47 °C). At the high AT (20 °C), the cumulative RR increased as the lag days increased and reached a maximum of 1.329 (95% CI 1.08–1.636) at lag 0–21.

**Table 2:** Cumulative relative risk (RR) of CVD hospital admissions at extreme AT during the lag days (reference 7.47 °C).

Extremely AT	Lag 0	Lag 0–3	Lag 0–7	Lag 0–14	Lag 0–21
Low (− 10 °C 5th)	0.747 (0.609–0.917)	1.044 (0.859–1.268)	0.979 (0.812–1.18)	0.762 (0.626–0.928)	0.75 (0.594–0.945)
High (20 °C 95th)	1.085 (0.962–1.224)	1.163 (1.027–1.317)	1.227 (1.055–1.429)	1.259 (1.048–1.512)	1.329 (1.08–1.636)

**Figure 2:**
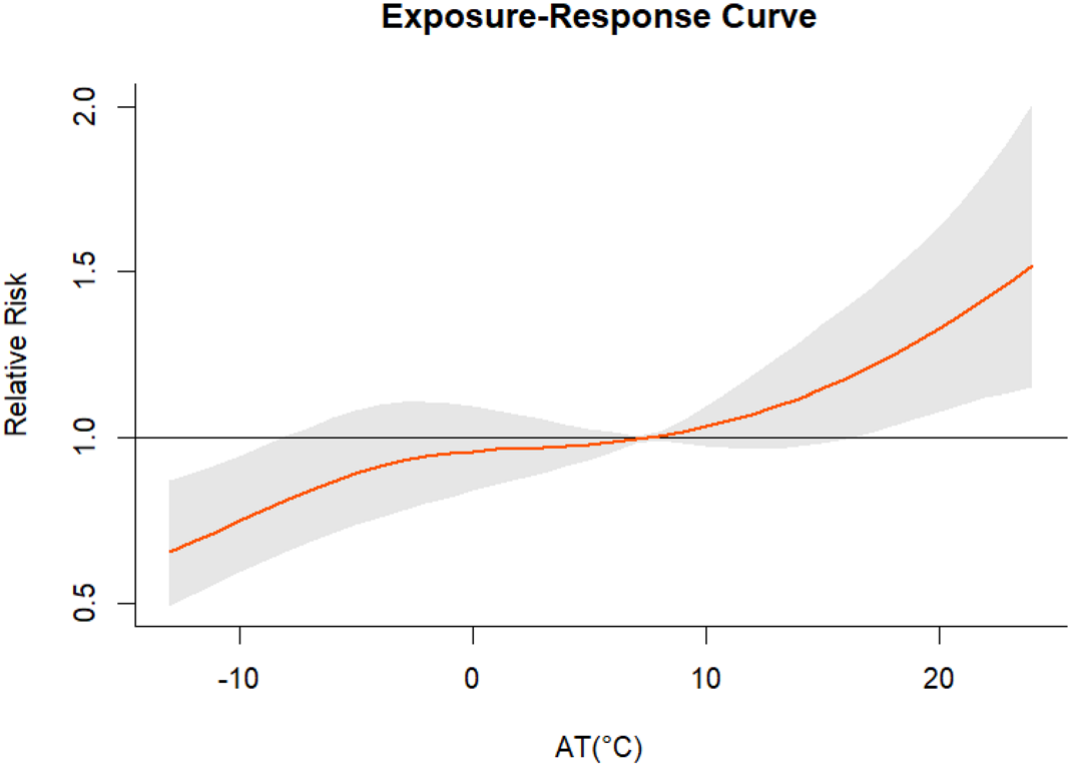
Exposure–response curve between AT and CVD hospital admissions in Linxia Autonomous Prefecture, China (reference 7.47 °C).

**Figure 3:**
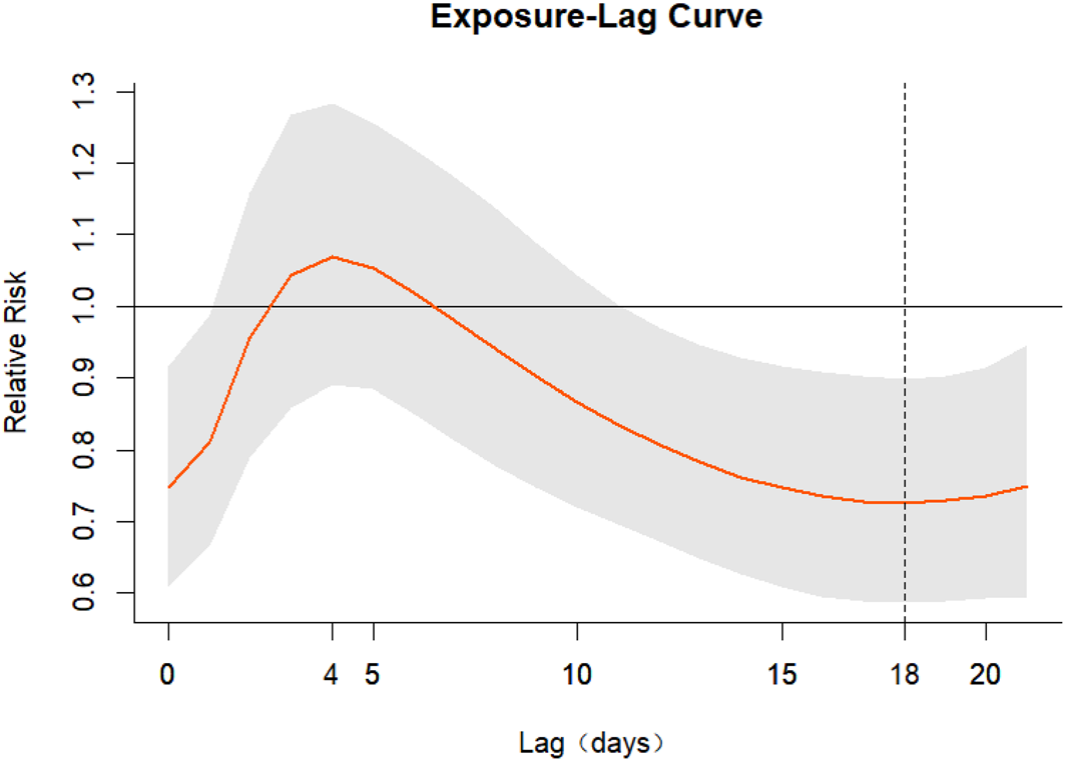
Exposure–lag curve between lag and CVD hospital admissions at AT = − 10 °C (reference 7.47 °C).

**Figure 4:**
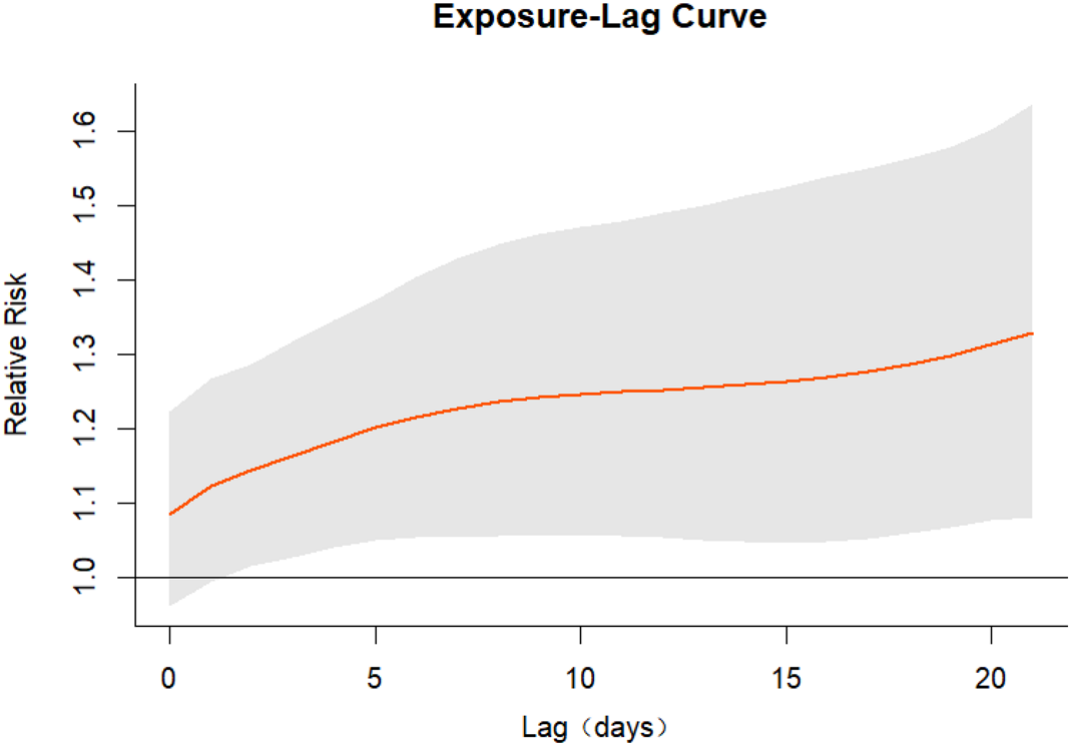
Exposure–lag curve between lag and CVD hospital admissions at AT = 20 °C (reference 7.47 °C).

Figure 2 is a cumulative exposure–response curve of AT on total CVD hospital admissions for over 21 lag days. As can be seen from Fig. 2, the curve keeps increasing with the increase in AT, and when AT is around − 10 °C, a protective effect was observed. When the AT was around 20 °C, there was a positive correlation between the number of admissions and AT. In addition, the gray area on the plot is the 95% CI. Regarding the low AT values (− 10 °C), the association between lag days and cumulative RR was an inverted U-shaped curve; the cumulative RR increased rapidly with the increase in lag days, then decreased at 4 lag days, and finally increased slightly at 18 lag days (Fig. 3). At high AT (20 °C), the RR increased with the increase in the number of lag days and reached the maximum in Fig. 4.

**Figure 5:**
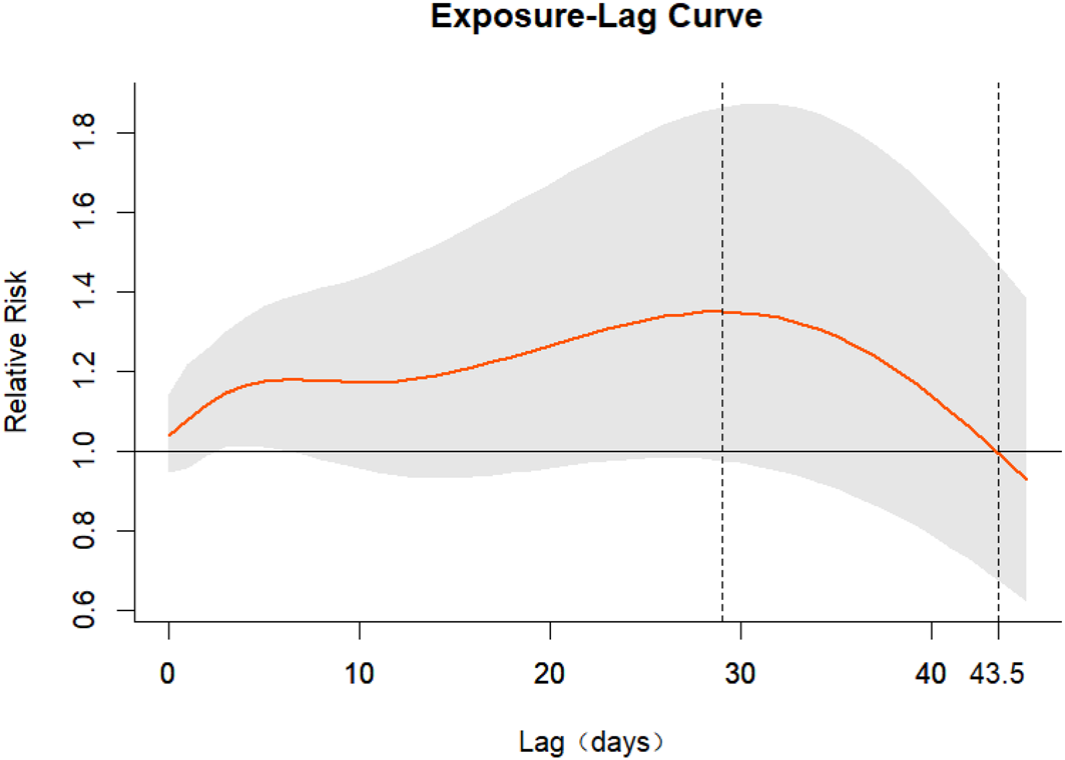
Exposure–lag curve between lag and CVD hospital admissions at AT = 20 °C (maximum lag = 45 days, reference 7.47 °C).

Based on Fig. 4, it is evident that the adverse impact of high AT persists at a maximum lag of 21 days. Therefore, we conducted an exploratory analysis where we extended the maximum lag to 45 days, as previous studies rarely exceeded a lag of 30 days. From Fig. 5, it can be observed that at AT = 20 °C, the cumulative RR reaches its maximum at a lag of 29 days. Subsequently, at a lag of 44 days, the cumulative RR falls below 1. At this point, the adverse effects of high AT have already gone. Although the results are not significant, it can be observed that high AT has a prolonged impact on the incidence of cardiovascular disease hospitalizations.

**Figure 6:**
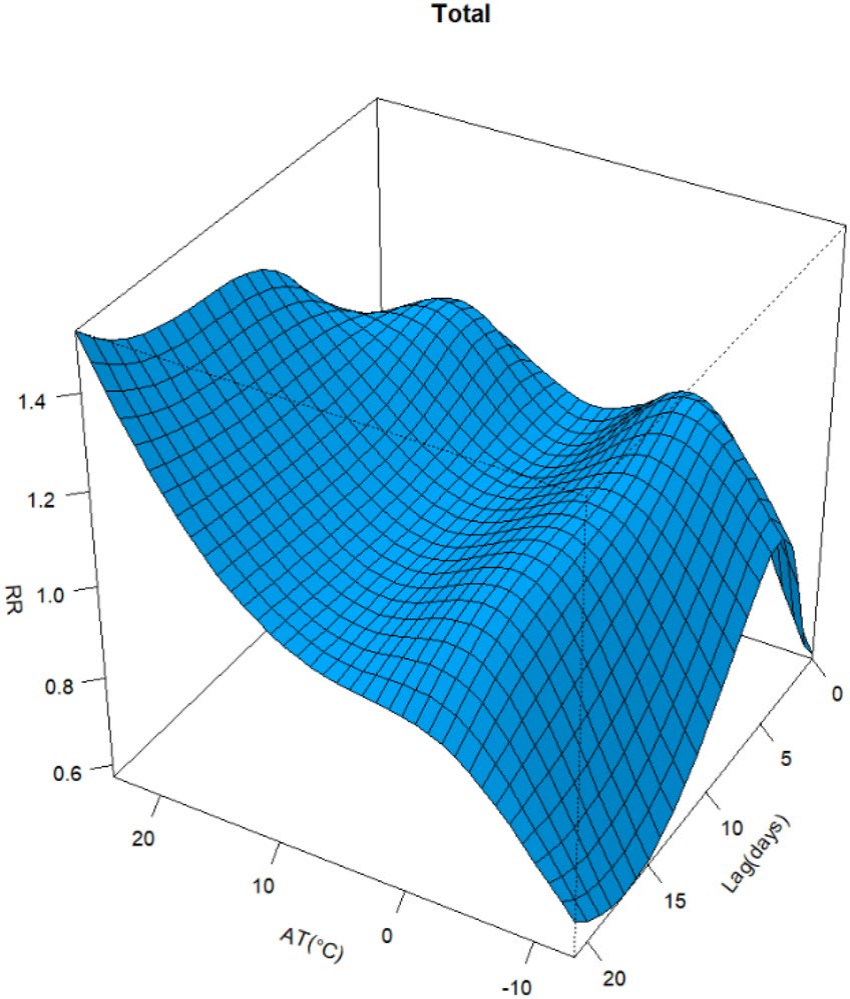
Three-dimensional plot of the association between AT and RR of CVD hospitalization over 21 lag days.

The relationship between AT and CVD hospital admission in different lag days is shown in Fig. 6, which is a three-dimensional (3D) plot of AT, RR, and lag days. There was a non-linear and lagged relationship between AT and the number of hospitalizations. The 3D figure shows that at lag 0–5 days, the cumulative RR was relatively high when the AT was low. At moderate AT, there was a slight change in the cumulative RR curve. The cumulative maximum RR value was 1.52 (95% CI 1.152–2.007) occurring at an AT of 24 °C and lag 21 days.

Table 3 shows the cumulative effect of low and high AT on CVD admissions in gender and age subgroups over 21 lag days. As can be seen from the gender group in the table, low AT was protective for males at lag 0 day, lag 0–14 and lag 0–21 days whereas for females, AT at lag 0–3 and lag 0–7 days was significantly associated with CVD admission. High AT had a significant relationship with CVD admissions in both males and females, and females were more affected than males. Low AT had a protective effect on the adults at lag 0–14 and lag 0–21 days, whereas high AT had a significant relationship with CVD hospitalizations in the elderly and adults, especially the adults, and the RR increased with the increase in lag days. Overall, females had a greater risk with high AT than males at lag 0–7, lag 0–14 and lag 0–21, adults had greater risk than the elderly at all lag days.

**Table 3:** Cumulative effect of low and high AT on CVD admissions in gender and age subgroups over 21 lag days.

	AT (°C)	Lag 0	Lag 0–3	Lag 0–7	Lag 0–14	Lag 0–21
Male	− 10	0.711 (0.521–0.971)	0.874 (0.65–1.174)	0.91 (0.692–1.218)	0.553 (0.409–0.747)	0.761 (0.534–1.084)
	20	1.098 (0.911–1.323)	1.204 (0.994–1.458)	1.157 (0.917–1.461)	1.192 (0.897–1.584)	1.173 (0.854–1.612)
Female	− 10	0.778 (0.594–1.019)	1.192 (0.921–1.544)	1.027 (0.801–1.317)	0.967 (0.746–1.255)	0.741 (0.545–1.007)
	20	1.073 (0.916–1.256)	1.136 (0.964–1.339)	1.283 (1.051–1.567)	1.312 (1.032–1.669)	1.457 (1.108–1.916)
Adults (< 65 years)	− 10	0.774 (0.573–1.047)	1.027 (0.771–1.369)	0.842 (0.639–1.109)	0.654 (0.489–0.875)	0.604 (0.428–0.851)
	20	1.171 (0.982–1.397)	1.395 (1.163–1.673)	1.404 (1.125–1.752)	1.544 (1.179–2.02)	1.773 (1.305–2.408)
Elderly (≥ 65 years)	− 10	0.727 (0.55–0.96)	1.055 (0.81–1.374)	1.113 (0.863–1.435)	0.864 (0.662–1.128)	0.898 (0.655–1.23)
	20	1.014 (0.86–1.197)	0.991 (0.835–1.176)	1.091 (0.885–1.343)	1.056 (0.821–1.357)	1.038 (0.783–1.377)

## Discussion

This study in Linxia Autonomous Prefecture, Gansu Province, China, used DLNM to analyze the cumulative effect of AT and lag on CVD admissions between January 2014 and December 2015. The analysis of the exposure–response curves revealed a non-linear relationship between AT and CVD admissions and showed that high AT had a persistent adverse effect on CVD admissions along the lag period. In the subgroups, females and adults were at greater risk with high AT than males and the elderly. Regarding low AT, there was a protective effect at lag 0 day, lag 0–14 days and lag 0–21 days.

The findings of the considerable effect of high AT on CVD admissions were consistent with the results of previous studies in Fujian^27^. A previous study in Tianshui, Gansu Province, China, found that the cumulative RR of CVD hospitalizations was 1.572 (95% CI 1.210–2.042) at lag 0–21 days for high AT, which was consistent with the cumulative RR of the present study^28^. Parliari et al.^29^ found that all-cause, cardiovascular, and respiratory mortality rates in Thessaloniki were mainly associated with high temperatures. Although the relationship between AT and mortality was not studied, mortality was associated with hospitalization. In Hong Kong, South China, Mohammad et al.^26^ suggested that the cumulative RR of CVD admissions increased at lag 0–28 days when temperature increased from 25 to 31.8 °C, which was consistent with the results of the present study showing an increased cumulative RR of admissions when exposed to high AT. This is attributed to the fact that exposure to high temperatures elevates heart rate, blood pressure, blood viscosity, and coagulation capability, while diminishing the capacity for core temperature regulation, thereby enhancing the susceptibility to cardiovascular disease^30^. Under high temperatures, vasodilation and blood flow redirection from vital organs to the skin surface for cooling purposes can potentially increase cardiac workload^31^. High temperatures can also induce physiological changes, such as increased blood viscosity and cardiac output, resulting in dehydration, hypotension, and even endothelial cell damage^8^. Indeed, these adverse effects have a prolonged duration, resulting in a long time lag for the impact of high temperatures.

Low AT had a protective effect on all lag days, except at lag 0–3 days and lag 0–7 days. At lag 0–3 days, the cumulative RR of CVD admissions was 1.044 (95% CI 0.859–1.268) for low AT (− 10 °C). These findings were similar to the results of a previous study of lag 0–3 days for extreme cold (2 °C)^28^. However, in other studies, the effect of cold seemed to be more harmful than that of heat. A previous study showed that low temperatures had delayed significant effects on CVD mortality^18^. The results of Silveira et al.^32^ from Brazil were inconsistent with the results of our study; they found a significant effect of cold and a protective effect of heat. In addition, Lu et al.^33^ found that the cumulative RR of CVD admissions had a significant association with low AT in Queensland, Australia. In low temperature conditions, the body undergoes various physiological changes, including alterations in blood pressure, vasoconstriction, increased blood viscosity, red blood cell count, elevated levels of plasma cholesterol and fibrinogen, ultimately leading to cardiovascular stress^34^. According to the study by Hong et al.^35^, the levels of high-density lipoprotein-cholesterol decline with decreasing temperature, which is considered as one of the significant contributing factors to the onset of cardiovascular diseases. This discrepancy could be attributed to many different factors. First, a previous study mentioned that when the temperature decreases, blood flow velocity increases, thereby enhancing blood circulation^36^. This would reduce the incidence of cardiovascular diseases. Second, the studies described were conducted in various parts of the world, with different climates and temperatures^37^. Linxia Hui Autonomous Prefecture is located in the Northwest of China, and its average temperature is lower than that of other regions. Therefore, the residents are more resistant to low temperatures and sensitive to high temperatures. Forth, living habits are different. More than 50% of Linxia people are ethnic minorities, and their eating and working habits are different from those in other places. For example, during the winter season, there is a decreased engagement in outdoor activities, specifically in agricultural practices, resulting in limited exposure to cold temperatures. As ethnic minorities, they often consume beef and mutton in their diet, both of which are rich sources of protein. A diet rich in protein from sources such as beef and mutton can provide additional energy and thermal regulation to withstand cold temperatures^28^. Therefore, this could account for the protective effect of low AT in Linxia. Fifth, the participants of our survey lived in rural Linxia, where there are no tall buildings or machine work, and there is more vegetation than in cities. Therefore, the average temperature in the countryside is lower than that in the city, and rural residents are more tolerant of cold. As a result, low AT had no effect on them, while high AT had a significant adverse effect^38^. Finally, Linxia has a higher altitude compared with some other plain areas in China. According to Sun et al.^39^, populations at low altitudes are more resistant to high temperatures, whereas people at high altitudes are more susceptible to heat effects.

In the current study, subgroup analyses by age and gender showed similar trends, with high AT having an adverse effect on CVD admissions. In the gender subgroup, heat adversely affected both sexes, but the effects were more persistent and severe for females. These results are consistent with those of Bühler et al.^40^, who suggested that females being more vulnerable to heat. The reasons for these findings may be that females are more susceptible to certain meteorological factors, or that males and females have different living habits^41^. However, Ponjan et al.^42^ and Wang et al.^28^ suggested that males were more affected by heat than females, possibly because males spend more time outdoors. Contrary to expectations, in the age subgroups, we found that adults (aged < 65 years) exposed to high AT were more adversely affected than the elderly (aged ≥ 65 years), and the cumulative RR in the adult group increased with increasing lag days. The findings are in line with the results that heatwaves had a significant effect on males and individuals aged 0–64 years in previous studies conducted in Vietnam^43^. In addition, Mohammadi et al.^44^ demonstrated that individuals aged ≤ 65 years were more susceptible to high temperatures than others in Tehran, Iran. A possible explanation for this might be that the heat tolerance of the elderly is higher than that of adults. Another possible explanation for this is that adults contribute to more outdoor jobs and activities and are more likely to be exposed to high temperatures. Contrary to the present results, many studies have demonstrated that heat has a greater impact on the elderly than on adults. Achebak et al.^45^ found that heat-related CVD mortality increased with age for both sexes. These results are in contrast to those of a study conducted in Tianshui, Gansu Province^28^. This inconsistency may be because the elderly have reduced sweat gland secretion and less redistribution of blood flow from the viscera and heart during heat exposure^46^.

In the current study, Poisson regression with DLNM was used to study the effect of extreme AT on CVD admissions in a rural population in Linxia Hui Autonomous Prefecture between January 2014 and December 2015. To the best of our knowledge, this is the first study to examine the relationship between AT and CVD admissions in the developing region of Linxia. We used AT as an indicator of the cold and hot effects to calculate the cumulative RR, which could more accurately represent the respondents’ sensitivity to the surrounding temperature. The results of this study can provide the local government with suggestions on the prevention strategies for CVD to reduce the effect of extreme temperatures on residents. In addition, the study had several limitations. Linxia Prefecture is inhabited by many ethnic minorities (59.2%), and their living habits and customs were not evaluated in the present study. Linxia is a relatively poor area, and some patients with mild CVD may not have presented to the hospital and may have presented to local clinics; therefore, these data may not be available.

## Conclusion

This study explored the relationship between extreme AT and cumulative RR of CVD admissions in Linxia Hui Autonomous Prefecture, Gansu Province, China. The high AT had a persistent adverse effect on residents, and the cumulative RR of CVD admission increased with increasing lag days. The high AT effect had more significant effects in females and adults than in males and the elderly. Although low AT had a protective effect on the lag 0 day and later lag days (lag 0–14 and lag 0–21 days). These findings can help the local government in devising preventive measures to reduce the number of CVD admissions and the adverse effect of extreme weather on the local population.

### Supplementary Information

Supplementary Information.

## Data Availability

The datasets generated during and/or analyzed during the current study are not publicly available but are available from the corresponding author on reasonable request.

## Abbreviations

AT: Apparent temperature
CVD: Cardiovascular diseases
DLNM: Distributed lag nonlinear model
RR: Relative risk of CVD
