# Supplementary material for: Effects of apparent temperature on cardiovascular disease admissions in rural areas of Linxia Hui Autonomous Prefecture
Source: Sci Rep. 2023 Sep 11;13:14971. doi: 10.1038/s41598-023-42232-9 (PMC10495458; doi:10.1038/s41598-023-42232-9)
Supplement: Supplementary file 1 — Supplementary Information. [file 41598_2023_42232_MOESM1_ESM.docx]

**Appendices:**

Fig S1 Sensitivity analysis of air pollutants


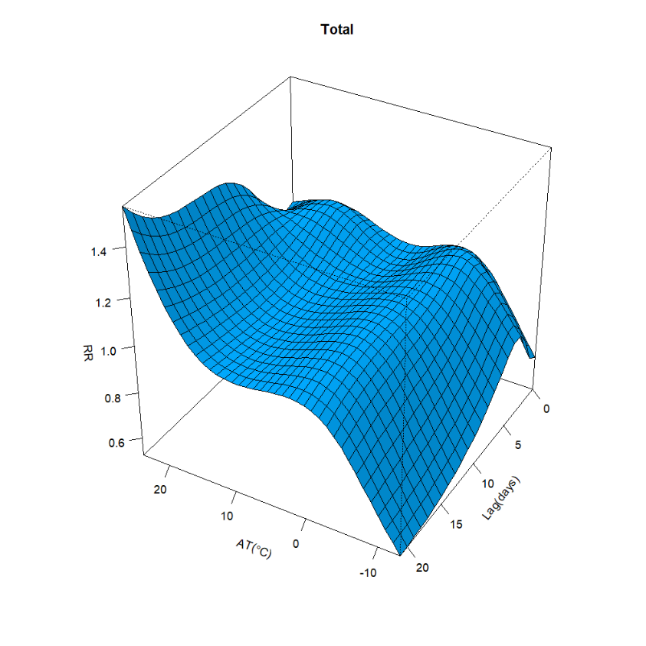

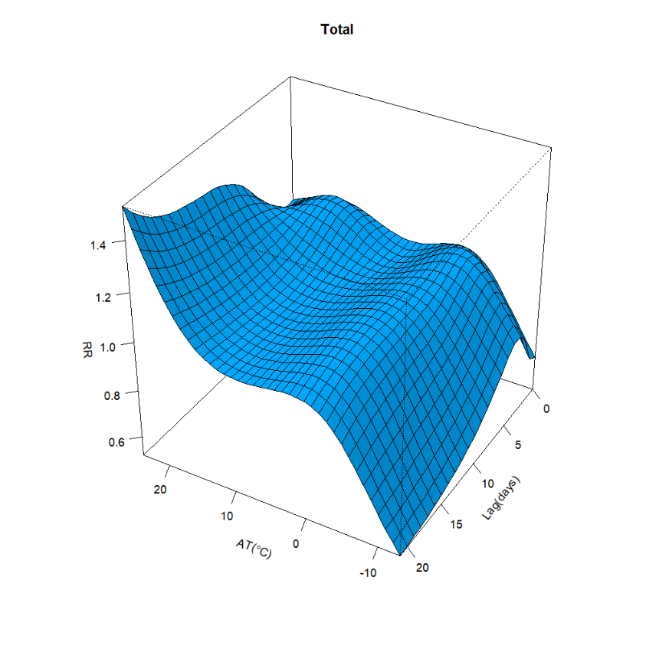

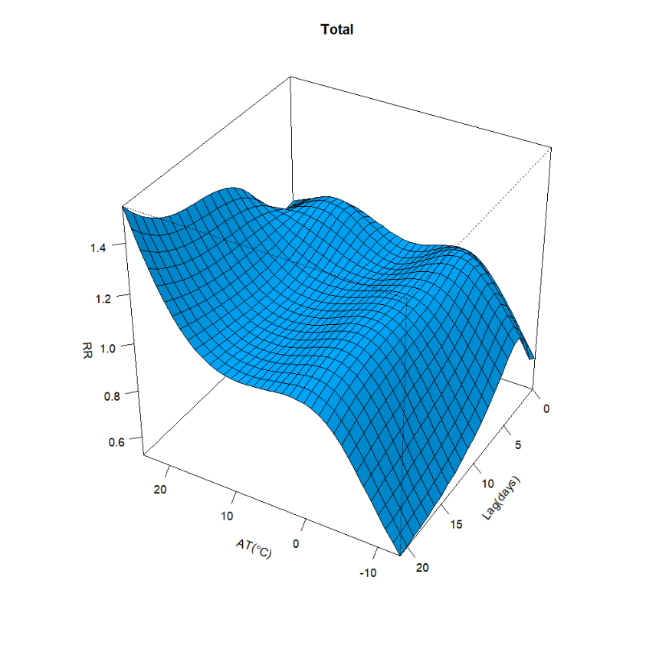


a sensitivity analysis on PM2.5 with 2 df a sensitivity analysis on PM2.5 with 3 df a sensitivity analysis on PM2.5 with 4 df


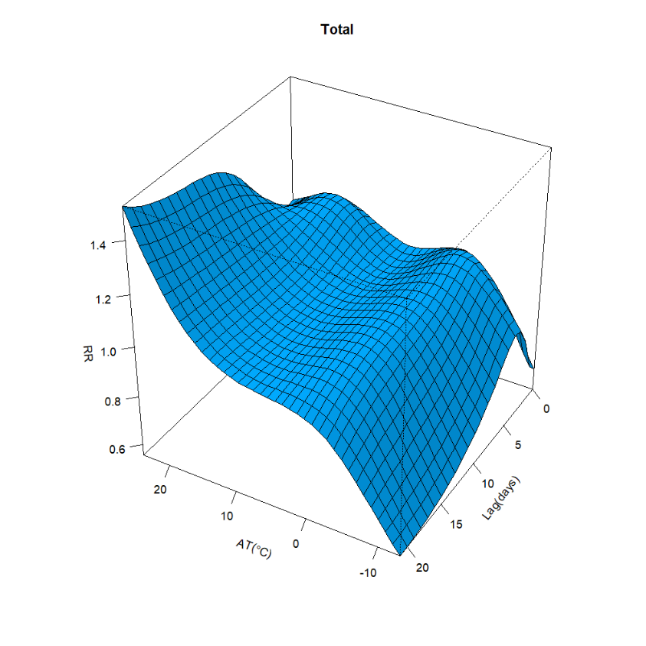

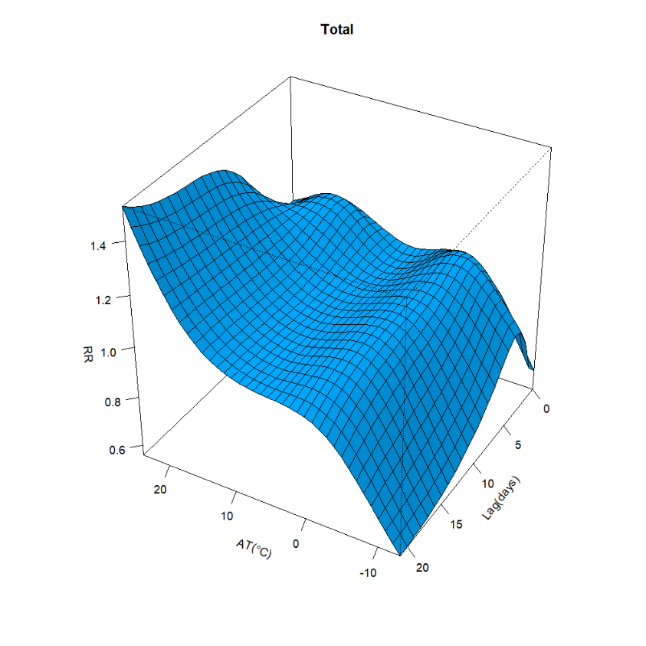

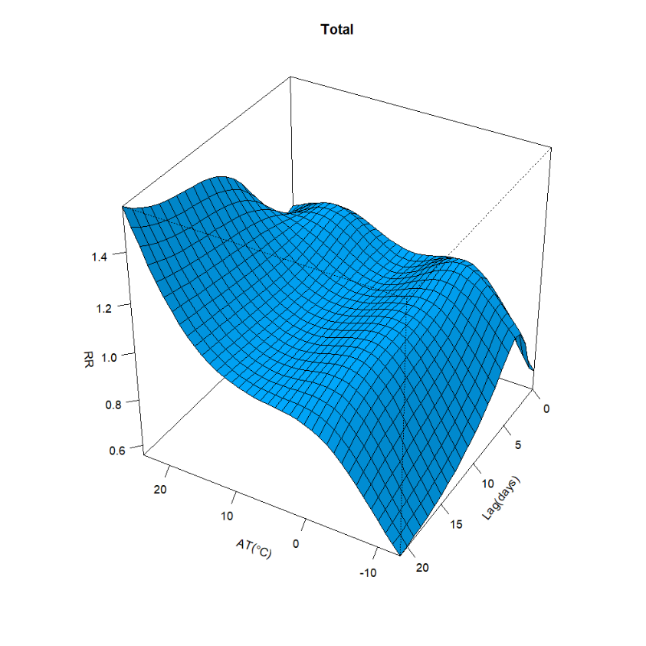


a sensitivity analysis on PM10 with 2 df a sensitivity analysis on PM10 with 3 df a sensitivity analysis on PM10 with 4 df

Fig S2 Sensitivity analysis was conducted with degrees of freedom of 6 for exposure response and 5 for exposure lag dimension.


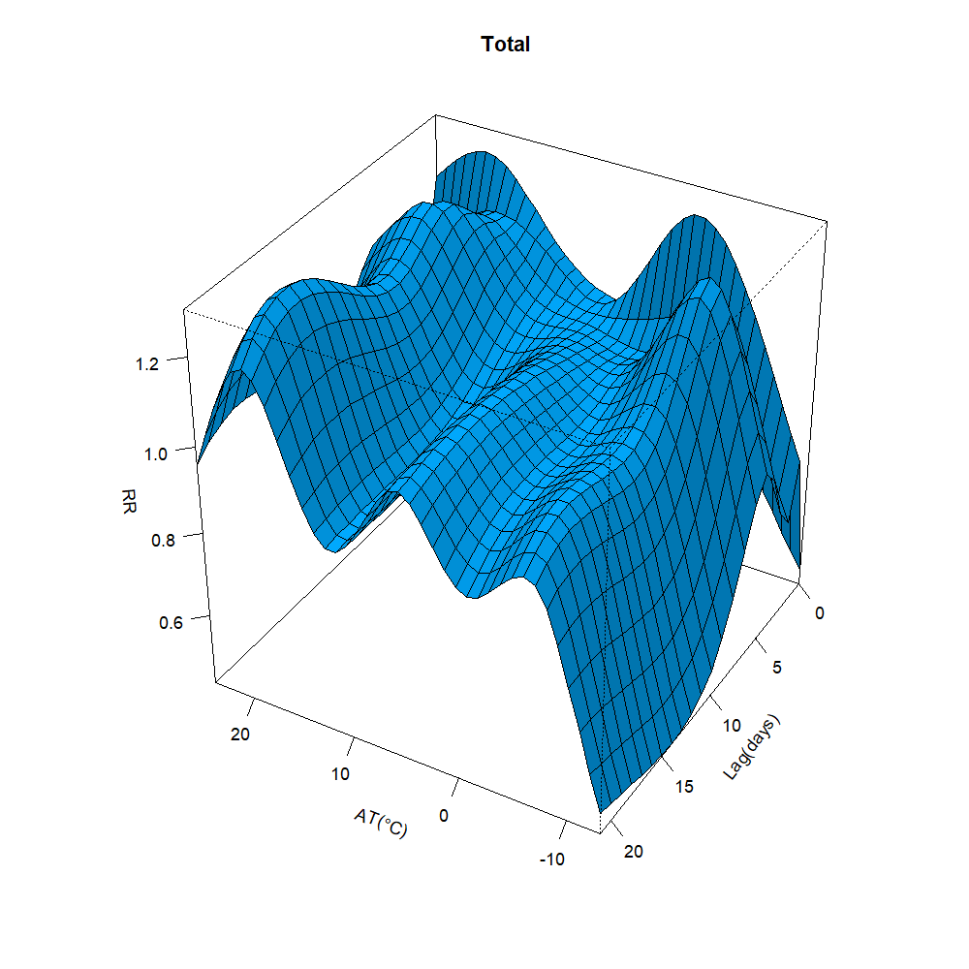


Table S1 The degrees of freedom for AT and lag in the cross-basis matrix were chosen according to the Akaike’s Information Criterion (AIC)

|  | df1 (exposure response） | df2 (exposure lag) | aic |
| --- | --- | --- | --- |
| 1 | 3 | 3 | 4616.003 |
| 2 | 3 | 4 | 4607.258 |
| 3 | 3 | 5 | 4601.498 |
| 4 | 3 | 6 | 4601.879 |
| 5 | 4 | 3 | 4589.145 |
| 6 | 4 | 4 | 4581.39 |
| 7 | 4 | 5 | 4576.635 |
| 8 | 4 | 6 | 4579.478 |
| 9 | 5 | 3 | 4592.746 |
| 10 | 5 | 4 | 4585.01 |
| 11 | 5 | 5 | 4581.772 |
| 12 | 5 | 6 | 4585.492 |
| 13 | 6 | 3 | 4581.523 |
| 14 | 6 | 4 | 4577.562 |
| **15** | **6** | **5** | **4574.04** |
| 16 | 6 | 6 | 4578.804 |
